# ACT-107, a novel variant of AmpC β-lactamase from *Enterobacter huaxiensis* isolated from Neotropical leaf frog (*Phyllomedusa distincta*) inhabiting the Brazilian Atlantic Forest

**DOI:** 10.1016/j.jgar.2023.04.016

**Published:** 2023-06

**Authors:** Johana Becerra, Gabriel G. Araujo, Felipe Vasquez-Ponce, Faride Lamadrid-Feris, Nilton Lincopan

**Affiliations:** a Department of Microbiology, Institute of Biomedical Sciences, University of São Paulo, São Paulo, Brazil; b One Health Brazilian Resistance Project (OneBR), São Paulo, Brazil; c Grupo Biodiversidad del Caribe Colombiano, Programa de Biología, Facultad de Ciencias Básicas, Universidad del Atlántico, Barranquilla, Colombia; d Department of Clinical Analysis, School of Pharmacy, University of São Paulo, São Paulo, Brazil

**Keywords:** AmpC β-lactamase, *Enterobacter* Spp., Antimicrobial resistance, Brazilian Atlantic Forest, Genomic surveillance

## Abstract

**Objectives:**

The aim of this study was to characterise a broad-spectrum cephalosporin-resistant AmpC-positive *Enterobacter huaxiensis* colonising the skin of a Neotropical frog (*Phyllomedusa distincta*) inhabiting the Brazilian Atlantic Forest.

**Methods:**

During a genomic surveillance study of antimicrobial resistance, we screened skin samples from *P. distincta*. Gram-negative bacteria growing on MacConkey agar plates supplemented with 2 µg/mL ceftriaxone were identified by matrix-assisted laser desorption/ionisation time-of-flight mass spectrometry. A cephalosporin-resistant *E. huaxiensis* was sequenced using the Illumina NextSeq platform. Genomic data were analysed using bioinformatics tools, whereas AmpC β-lactamase was characterised in depth by comparative analysis of amino acids, *in silico* modelling, and analysis of susceptibility to β-lactam antibiotics and combinations of β-lactamase inhibitors.

**Results:**

Whole-genome sequencing analysis revealed a novel variant of AmpC β-lactamase belonging to the ACT family, designated ACT-107 by NCBI. This variant contains 12 novel amino acid mutations within the ACT family, 5 in the signal peptide sequence (Ile2, Met14, Tyr16, Gly18 and Thr20), and 7 in the mature protein (Gln22, His43, Cys60, Thr157, Glu225, Ala252 and Asn310). *In silico* modelling showed that substitutions occurring in the mature chain are localised in the solvent-accessible surface of the protein, where they are not expected to affect the β-lactamase activity, as observed in the resistance profile. Strikingly, ‘not designated’ ACT variants from *E. huaxiensis* were clustered (> 96% identity) with ACT-107.

**Conclusion:**

Since *E. huaxiensis* has been isolated from human infection, ACT-107 requires surveillance and the attention of clinicians.

## Highlights

• In silico characterisation of a novel AmpC variant from *E. huaxiensis* is presented.
• The variant confers resistance to broad-spectrum cephalosporins and cephamycins.
• ACT-107 AmpC displays 12 unique mutations within the ACT family.
• Frogs may represent important hosts of emerging species with novel β-lactamases.

## 1 Introduction

Bacteria of the *Enterobacter* genus are widely distributed in the environment (e.g., in drinking water, sewage, and soil), and frogs have been reported to be natural hosts [1,2]. In the last few decades, this genus has taken on clinical significance as nosocomial pathogens associated with urinary tract infections, respiratory infections, soft tissue infections, osteomyelitis, and endocarditis, among others [1,3]. Moreover, genomic approaches have allowed the identification of novel and emerging species, including *Enterobacter huaxiensis*, which was proposed in 2019 [4].

The clinical significance of *Enterobacter* lies in its ability to acquire multiple resistance determinants to antibiotics used in therapy. In this regard, some *Enterobacter* species carry intrinsic AmpC-type β-lactamase genes (i.e., *bla*_ACT-type_) [1,5]. AmpC β-lactamases belong to class C Ambler's classification. Structurally, AmpC enzymes consist of α and α/β domains, where a serine residue in the active site catalyses acylation and deacylation reactions, as a mechanism of enzymatic inactivation of antibiotics [6], [7], [8]. These enzymes are clinically important, since they can hydrolyse penicillins and narrow- and broad-spectrum cephalosporins. Some variants display weak hydrolytic activity against carbapenems (e.g., CMY-2, ADC-68, and ACT-1) [8,9]. Since some AmpC β-lactamases occur on transmissible plasmids, they constitute a risk for the dissemination of multidrug-resistance genes. To date, 101 variants of the ACT family have been reported [10]. We hereby report ACT-107, a novel AmpC variant identified in an *E. huaxiensis* strain isolated from the skin of an endemic Neotropical frog (*Phyllomedusa distincta*) inhabiting the Brazilian Atlantic Forest.

## 2 Materials and methods

### 2.1 Bacterial identification and antimicrobial susceptibility testing

During a genomic surveillance study conducted to monitor the occurrence of clinically relevant antimicrobial-resistant Enterobacterales in amphibians, skin swab samples were collected from 11 adults of *Phyllomedusa distincta*, inhabiting an Atlantic Forest reserve (Parque Estadual Intervales, 24° 16′ 06.3" S 48° 24′ 49.7" W), in São Paulo, Brazil. The frogs were captured in November 2017, in a locale surrounded by a large area of primary forest, near ecotourism activities. To efficiently remove transient bacteria from the skin, all frogs were rinsed with sterile water before swabbing their dorsal, ventral, and lateral sides [11]. Skin swabs were plated on R2A agar (Difco) and incubated at room temperature for 48 h. To select broad-spectrum cephalosporin-resistant bacteria, different colonies grown on R2A agar were sub-cultured on MacConkey agar (Difco) plates supplemented with 2 µg/mL ceftriaxone (Sigma). Next, bacterial identification was performed using matrix-assisted laser desorption/ionisation time-of-flight mass spectrometry (Microflex, Bruker Daltonik, Germany).

Antimicrobial susceptibility was determined using the disk-diffusion method on Mueller–Hinton agar (Difco) [12,13]. Antibiotics (µg/disk) tested included aztreonam (30), ampicillin (10), amoxicillin (10), piperacillin (30), ticarcillin (75), ampicillin/sulbactam (10/10), amoxicillin/clavulanic acid (20/10), piperacillin/tazobactam (30/6), ceftolozane/tazobactam (30/10), ticarcillin/clavulanic acid (75/10), cefoxitin (30), cephalothin (30), cefaclor (30), cefuroxime (30), cefoperazone (30), cefotaxime (30), ceftazidime (30), ceftazidime/avibactam (14 and 50), ceftriaxone (30), ceftiofur (30), cefpodoxime (10), cefepime (30), doripenem (10), imipenem (10), ertapenem (10), meropenem (10), and cefiderocol (30). Interpretative criteria were based on the EUCAST and CLSI guidelines [12,13]. *Escherichia coli* ATCC 25922 was used as a control for the antimicrobial susceptibility tests. Antibiotic disks were provided by Cefar Diagnóstica Ltda (São Paulo, Brazil) or purchased from Liofilchem (Roseto D.A., Italy).

AmpC production was confirmed via the disk potentiation method using 3-amino phenyl boronic acid (APB). Cefoxitin (30 µg/disk) and cefoxitin/APB (30/300 µg/disk) were used [14]. The agar plates were incubated at 37°C overnight, and the diameter of the inhibition zones was compared for the detection of class C β-lactamases. Additionally, AmpC induction was investigated by the disk approximation (D-test) method, as previously described [15], using ceftazidime/avibactam (CZA; 10/4 µg), cefiderocol (30 µg), and cefepime (30 µg) as substrates and imipenem (10 µg) as an inducer.

### 2.2 Whole-genome sequencing (WGS) analysis

Genomic DNA of *E. huaxiensis* isolate 4Pd9 was extracted using the PureLink Quick Gel Extraction Kit (Life Technologies, USA), and a genomic paired-end library was prepared using a Nextera DNA Flex Library Preparation Kit (Illumina Inc., UK), according to the manufacturer's instructions. The whole genome was sequenced on the Illumina NextSeq platform (Illumina Inc., CA). Reads were de novo assembled using Unicycler v.0.4.8, and automatic annotation was performed using Prokka v.1.13.3 [16,17]. Average nucleotide identity (ANI) was used to determine the identity of the 4Pd9 bacterial species, using ANI values of >95% and >98% as thresholds for species and subspecies definitions, respectively [18,19]. The genomes were evaluated using the ResFinder v.4.1 tool from the Centre for Genomic Epidemiology [20] and the Comprehensive Antibiotic Resistance Database [21]. The genetic context of *bla*_ACT-107_ was analysed with the EFI–Genome Neighborhood tool, using the BLAST algorithm (https://efi.igb.illinois.edu/efi-gnt/).

A phylogenetic tree analysis of ACT-type sequences was performed based on representative sequences retrieved from the Beta-Lactamase DataBase and GenBank database. Analysis was performed using MEGA v.10.1.5 [10,22]. Amino acid sequences were aligned using ClustalW, and a neighbor-joining tree was generated with 1000 bootstrap replicates. The same alignment was used to identify unique mutations in the ACT-107 sequence in relation to all other ACT-type proteins. Tree topology visualisation and annotation were performed with iTol v.6 [23].

### 2.3 In silico modelling and alignment of representative ACT-type protein sequences

The ColabFold implementation of the AlphaFold2 software was used to generate a prediction of the ACT-107 tertiary structure with high confidence (98 average predicted local distance difference test score and 0.96 predicted template modelling score) [24]. The model was then aligned with a previous AmpC structure solved in the acyl-enzyme state with ceftazidime (DOI: 10.2210/pdb1IEL/pdb, PDB ID: 1IEL; root mean square deviation, 0.373 Å; 71% sequence identity) [25]. The ligand coordinates were merged with the ACT-107 model, and the covalent bond with the catalytic serine was manually created before energy minimisation of the complex with the Yasara server [26]. LigPlot^+^ v.2.2 was used to compare ligand-protein interactions between the model and AmpC (1IEL) [27]. Structural analyses were performed using PyMOL (https://pymol.org/2/) and ChimeraX [28].

Amino acid sequences of publicly available ACT-type β-lactamases, including ACT-10, were clustered with CD-HIT [29], using a sequence identity cut-off of 0.95 to extract a total of six representative sequences. The sequences were aligned using Clustal Omega v.1.2.4 [30] and rendered with the predicted secondary structure elements of the ACT-107 AlphaFold2 model using ESPript v.3.0.20 [31]. SignalP v.6.0 was used to predict the signal peptide in the sequences [32].

## 3 Results and discussion

In this study, we identified two *Enterobacter* Spp. (strains 4Pd7 and 4Pd9) displaying resistance to broad-spectrum cephalosporins as colonisers of Neotropical leaf frogs. Whereas strain 4Pd7 was identified as *E. asburiae*, carrying the *bla*_ACT-4_ ampC gene and previously described as intrinsic to this species (GenBank accession number: EU427302), strain 4Pd9 was confirmed by genomic ANI analyses to be *E. huaxiensis* with 99.1% nucleotide identity to the reference sequence CP043342.1. Notably, this strain carried a novel ACT-type AmpC and displayed resistance to ampicillin, amoxicillin, piperacillin, ticarcillin, amoxicillin/clavulanic acid, ampicillin/sulbactam, piperacillin/tazobactam, ticarcillin/clavulanic acid, cefoxitin, cephalotin, cefaclor, cefuroxime, cefoperazone, cefotaxime, ceftazidime, ceftriaxone, ceftiofur, and cefpodoxime. In addition, *E. huaxiensis* 4Pd9 exhibited intermediate susceptibility to imipenem and doripenem, remaining susceptible to aztreonam, cefepime, ceftolozane/tazobactam, ceftazidime/avibactam, ertapenem, meropenem, and cefiderocol (Table 1). The strain 4Pd9 exhibited a positive AmpC induction test with the CZA/imipenem and cefiderocol/imipenem combination.

**Table 1:** β-lactam susceptibility profile of *Enterobacter huaxiensis* strain 4Pd9 carrying the ACT-107 AmpC variant.

β-lactam antibiotics	Interpretative criteria	
	CLSI	EUCAST
Ampicillin	R	R
Amoxicillin	R	R
Aztreonam	S	S
Piperacillin	R	R
Ticarcillin	R	R
Ampicillin/sulbactam	R	R
Amoxicillin/clavulanic acid	R	R
Piperacillin/tazobactam	R	R
Ticarcillin/clavulanic acid	R	R
Ceftolozane/tazobactam	S	S
Cephalothin	R	R
Cefaclor	R	R
Cefuroxime	R	R
Cefoperazone	R	R
Cefotaxime	R	R
Ceftriaxone	R	R
Ceftiofur	R	R
Ceftazidime	R	R
Cefoxitin	R	R
Cefpodoxime	R	R
Cefepime	S	S
Ceftazidime/avibactam	S^a^	S^b^
Doripenem	I	I
Imipenem	I	I
Ertapenem	S	S
Meropenem	S	S
Cefiderocol	S	S

a Ceftazidime/avibactam (30 µg/20 µg).

b Ceftazidime/avibactam (10 µg/4 µg).

WGS analysis of *E. huaxiensis* 4Pd9 revealed the presence of a novel chromosome-encoded AmpC variant of the ACT family, designated ACT-107 (GenBank accession number: UNN26045) by NCBI. Since no other β-lactamase was identified in the genome of 4Pd9, ACT-107 was assumed to be responsible for the resistance profile to penicillins, narrow- and broad-spectrum cephalosporins, cephamycins, and β-lactam/β-lactamase inhibitor combinations. No plasmids were predicted from the WGS analysis of *E. huaxiensis* 4Pd9.

The *bla*_ACT-107_ gene is flanked by *ampR* (regulator) and *blc* genes (Fig. S1). The sequences of *ampR, ampG*, and *ampD* were also analysed and compared with the *ampR, ampG*, and *ampD* sequences from *E. cloacae* (Uniprot Taxon ID 550), sharing 89.69%, 91.85% and 90.91% identity, respectively. Although mobile genetic elements were screened, no plasmids were predicted from the WGS analysis of *E. huaxiensis* 4Pd9; indeed, the *bla*_ACT-107_ gene was inserted in a conserved region of the chromosome, as confirmed by using the Genome Neighborhood tool (Fig. S1).

**Fig. 1:**
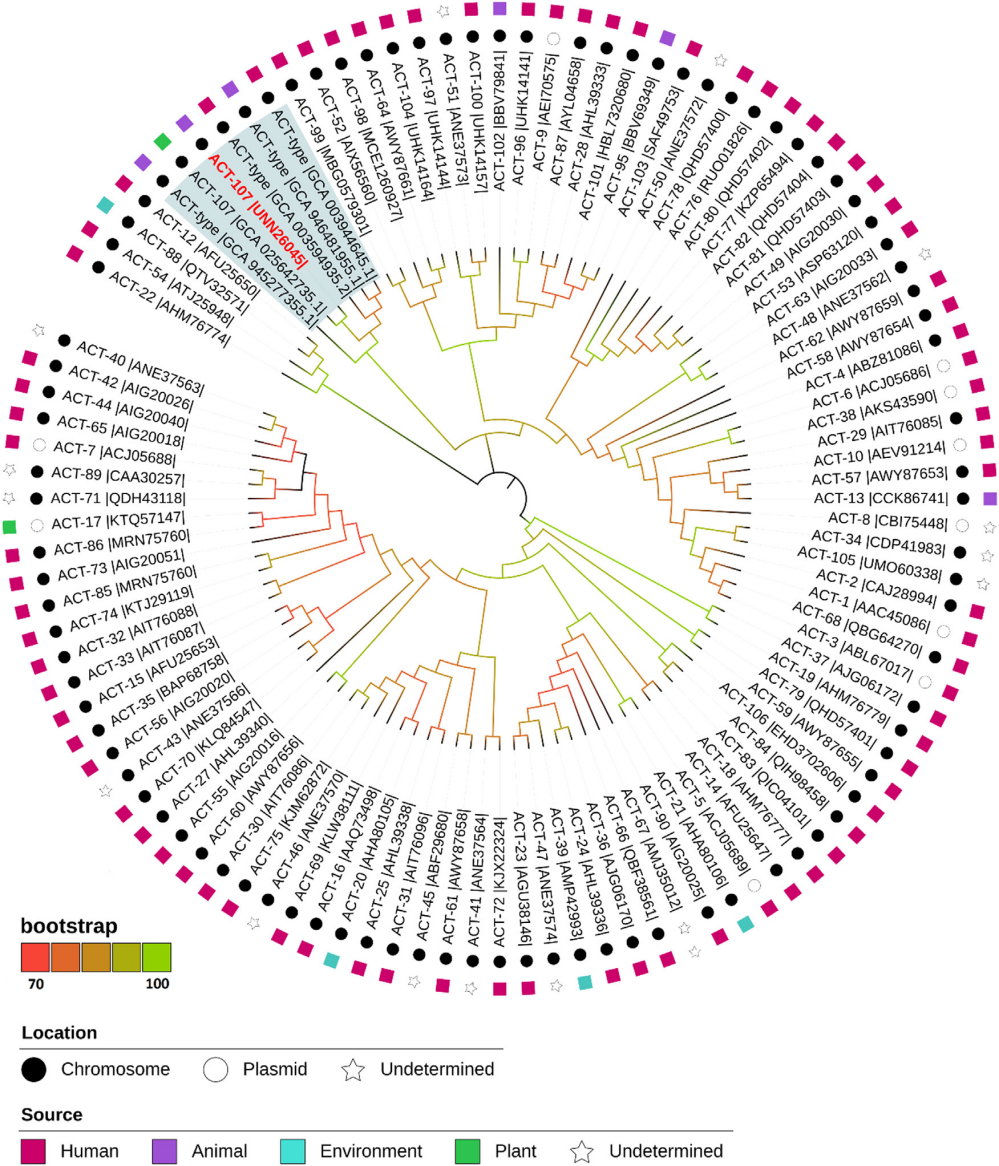
Phylogenetic tree based on a comparative analysis of ACT-type amino acid sequences retrieved from the β-lactamase database. The ACT-107 (this study) sequence is highlighted in red. Four novel unpublished and not designated ACT variants from *Enterobacter huaxiensis*, sharing 96.5% (GenBank accession number: GCA_003594935.2), 97.6% (GenBank accession number: GCA_946481955), and 98.9% (GenBank accession numbers: GCA_003944645.1 and GCA_945277355.1) identity with ACT-107, and the ACT variant from the strain RTI-PI-AC (GenBank accession number: GCA_025642735.1), isolated from poison ivy in United States of America, sharing 100% identity with ACT-107, are in blue shadow. The tree was generated using the neighbor-joining method with 1000 bootstrap replicates in MEGAX and tree topology in iTOL v6.1.2. Next to each branch, the source of the variant is shown according to NCBI entry data.

**Fig. 2:**
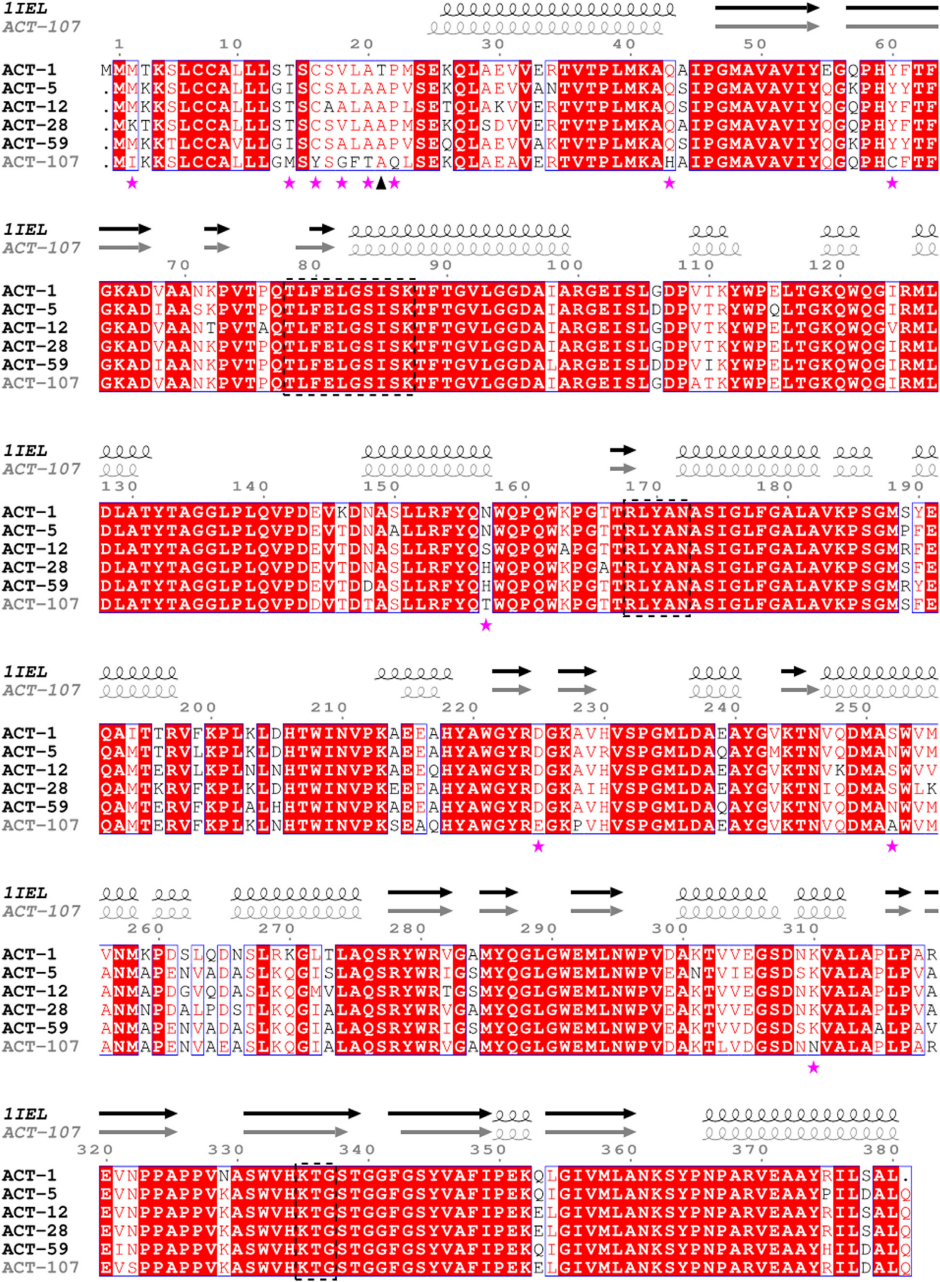
Alignment of representative amino acid sequences of ACT-type β-lactamases: ACT-1 (GenBank accession no. AAC45086), ACT-5 (ACJ05689), ACT-28 (AHL39333), ACT-59 (AWY87655), ACT-12 (AFU25650), and ACT-107 (UNN26045, this study). On top of aligned sequences, α-helix (spiral regions) and β-pleated sheets (arrows) of ACT-107 were predicted based on the crystal structure of AmpC (PDB ID: 1IEL) β-lactamase from *Escherichia coli*. Conserved amino acid motifs of AmpC β-lactamases are surrounded by dotted boxes. Mutations found in ACT-107 are highlighted with stars, and an arrowhead denotes the first amino acid of the mature proteins after processing by Signal Peptidase I. ACT-1, ACT-5, ACT-12, ACT-28, and ACT-59 variants were selected for alignment, based on cluster analysis using CD-HIT [29], in order to select representative ACT variants sharing 85–87% identity with ACT-107.

A phylogenetic tree of all ACT-type β-lactamase sequences from *Enterobacter* species was created to compare the identity of amino acid sequences of ACT variants against the novel ACT-107 (Fig. 1). This alignment revealed 12 unique amino acid substitutions in ACT-107, 5 in the signal peptide sequence (Ile2, Met14, Tyr16, Gly18, and Thr20), and 7 in the mature protein (Gln22, His43, Cys60, Thr157, Glu225, Ala252, and Asn310) (Fig. 2). Curiously, while most ACT-type variants were clustered within groups sharing high amino acid identities, ACT-107 and ACT-50 were found to be relatively distant from previously described ACT variants (Fig. 1). Interestingly, analysis of four *E. huaxiensis* genomes, recently submitted to NCBI, revealed the presence of undesignated ACT variants, sharing 96.5% (GenBank accession number: GCA_003594935.2), 97.6% (GenBank accession number: GCA_946481955), and 98.9% (GenBank accession numbers: GCA_003944645.1 and GCA_945277355.1) amino acid identity with the novel ACT-107 variant, whereas the ACT variant from the strain RTI-PI-AC (GenBank accession number: GCA_025642735.1), isolated from poison ivy in the United States of America, shared 100% identity with ACT-107 (Fig. S2). Therefore, most likely, isolates belonging to the *E. huaxiensis* species carry phylogenetically related ACT-107–type variants, which could be a marker for the precise identification of the species.

**Fig. 3:**
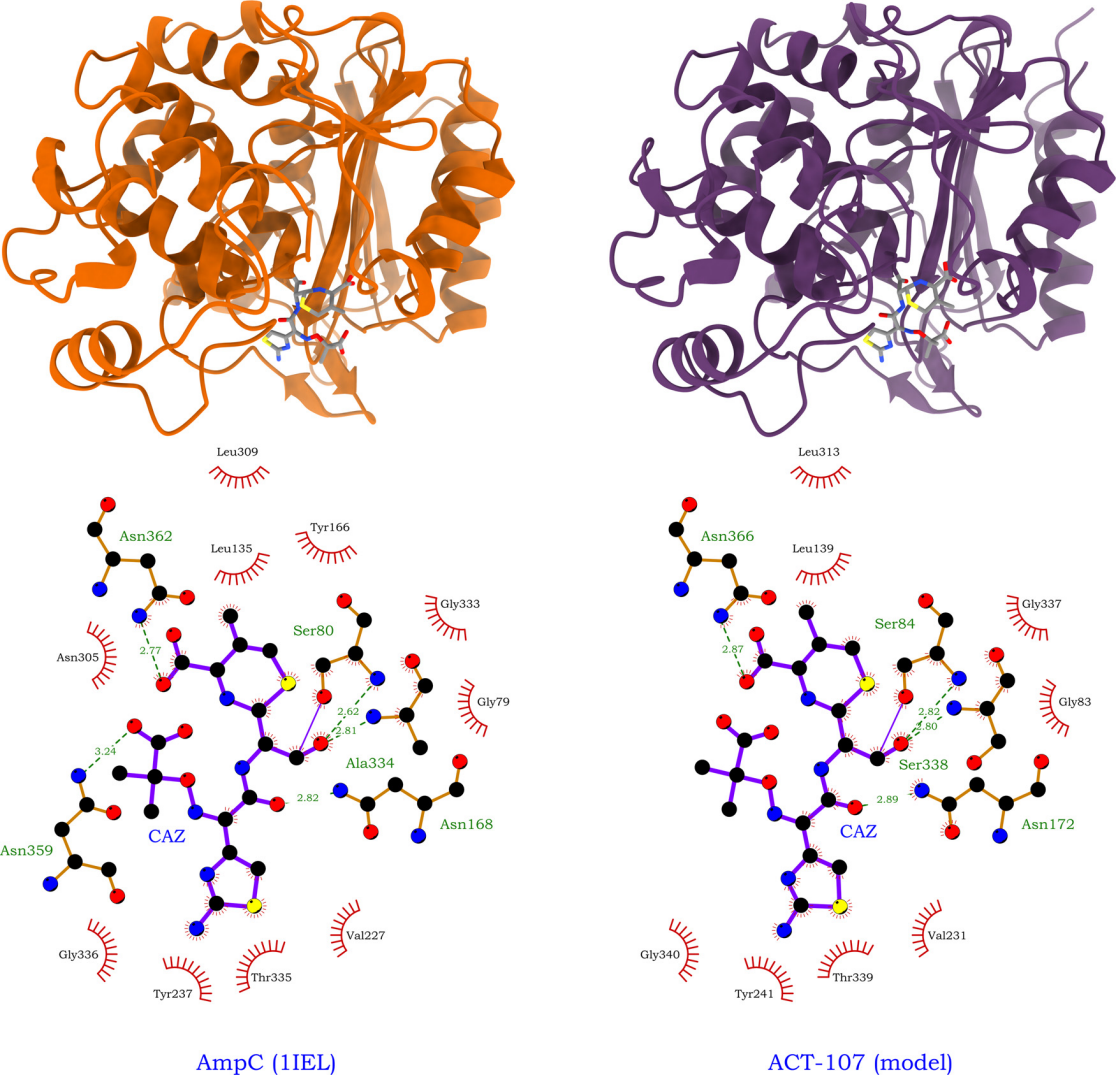
In silico comparison of the acyl-enzyme complex formed by ceftazidime and AmpC β-lactamases. On the top left, a model of the AmpC enzyme 1IEL from *Escherichia coli* (PDB DOI: 10.2210/pdb1IEL/pdb) is shown, displaying an energy-minimised structure. On the top right, an AlphaFold2 model of ACT-107 AmpC β-lactamase from *Enterobacter huaxiensis* strain 4Pd9, bound to ceftazidime, is shown. On the bottom left and right, LigPlot^+^ diagrams representing specific ceftazidime-enzyme interactions of the AmpC 1IEL model structure and the ACT-107 AmpC β-lactamase are shown, respectively.

The in silico modelling of ACT-107 bounded to acylated ceftazidime showed that this variant is able to accommodate and hydrolyse oxyimino-cephalosporins of clinical relevance (Fig. 3), in accordance with the observed phenotypic profile (Table 1). The signal peptide of the protein was predicted with a high confidence (0.99) by the SignalP server, indicating that the five mutations in this sequence are not expected to affect protein secretion to the periplasm. Additionally, the seven mutations in the mature chain are predicted to localise away from the ligand pocket (Fig. S3), in the solvent-exposed surface of the AlphaFold2 model, and thus are not expected to contribute to modifications in the enzyme performance, as supported by the β-lactam resistance profile (Table 1).

In concordance with AmpC serine β-lactamases, the ACT-107 enzyme contained all conserved motifs (i.e., T[LI]F[ED][LIV]GS[VIL]SK, RxYxN, and KTG) responsible for catalytic activity and/or substrate binding (Fig. 2) [9]. TheLigPlot^+^ diagram revealed that the ACT-107/ceftazidime complex model and the AmpC 1IEL show an analogous ligand orientation and a similar pattern of hydrogen bonds and hydrophobic interactions (Fig. 3). For ACT-107, hydrogen bonds from Asn172, Asn366, and Ser338 side chain amino acids appear to be important to stabilise the ligand in the pocket. Indeed, these amino acids appear to be functionally equivalent to Asn168, Asn362, and Ala334 from AmpC 1IEL (Fig. 3). Therefore, no change in the enzyme-substrate binding mechanism of ACT-107 is expected, in comparison with that of classical AmpC β-lactamases.

Distinguishing β-lactamases in Enterobacterales has epidemiological significance. In this regard, some studies have demonstrated the contribution of *Kluyvera, Citrobacter*, and *Enterobacter* species to the recruitment and dissemination of plasmid-mediated extended-spectrum β-lactamases and/or AmpC β-lactamases [8,[33], [34], [35], [36]]. Specifically, the presence of AmpC in plasmids has contributed to the rapid spread of this mechanism of resistance.

Plasmid-mediated AmpC (pAmpC) genes have originated from chromosomal *ampC* genes carried by several Gram-negative species and are classified into at least five phylogenetic groups: *Enterobacter* (MIR, ACT), *Citrobacter freundii* (CMY-2-like, LAT, CFE), *Morganella morganii* (DHA), *Aeromonas* group (CMY-1like, FOX, MOX), and *Hafnia alvei* (ACC) [8,36]. Therefore, knowing the distribution of ACT-type β-lactamases in *Enterobacter* species will allow inferences regarding the putative origin of pAmpCs that could emerge in clinically relevant pathogens. In this respect, the chromosomally encoded AmpC β-lactamase reported in *E. hormaechei* has been suggested as the putative progenitor of the pACT-1 found in *Klebsiella pneumoniae* [34,37].

A limitation of this study was the lack of an enzymatic kinetics assays. On the other hand, although mutations in *ampR, ampG*, and *ampD* were identified [38], transcriptional regulation mechanisms have been not investigated. However, the ACT-107 AmpC activity could be evidenced by the resistance phenotype to penicillins, broad-spectrum cephalosporins, cephamycins, and β-lactam/β-lactamase inhibitor combinations (Table 1).

Next-generation sequencing technologies and in silico analysis of antimicrobial resistance genes from bacterial genomes has allowed tracking of the origin and successful expansion of clinically relevant resistance mechanisms. For accurate analyses, user-friendly and publicly available bioinformatic tools based on versions of databases must be constantly updated. Therefore, it is crucial that novel resistance genes, including the AmpC variant, are identified and published, in order to keep antimicrobial resistance gene repositories as up to date as possible [39], [40], [41].

In summary, this study highlights several important issues. First, we reported for the first time the identification of a novel ACT AmpC variant, ACT-107, that confers resistance to penicillins, broad-spectrum β-lactams, and β-lactam/β-lactamase inhibitor combinations, in an *E. huaxiensis* strain colonising an amphibian host. Second, phylogenetically related ACT-107–type variants seem to be restricted to the *E. huaxiensis* species. Third, since *E. huaxiensis* has been isolated from human infections [4], the clinical significance of ACT-107 requires evaluation and the attention of clinicians.

## Competing interests

None declared.

## Appendix Supplementary materials

Image, application 1
