# Supplementary material for: ACT-107, a novel variant of AmpC β-lactamase from Enterobacter huaxiensis isolated from Neotropical leaf frog (Phyllomedusa distincta) inhabiting the Brazilian Atlantic Forest
Source: J Glob Antimicrob Resist. 2023 Jun;33:353–9. doi: 10.1016/j.jgar.2023.04.016 (PMC10275763; doi:10.1016/j.jgar.2023.04.016)
Supplement: Supplementary file 1 [file mmc1.docx]

**Supplementary material**

**
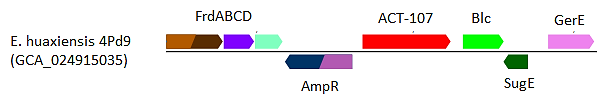
**

**Figure S1**

**Fig S1.** Genetic context of *bla*_ACT-107_ in *E. huaxiensis* 4Pd9 strain. The *bla*_ACT-107_ gene is flanked by the transcriptional regulator AmpR and the outer membrane lipoprotein Blc. Open reading frames are shown with their directions of transcription indicated by broad arrows.

**
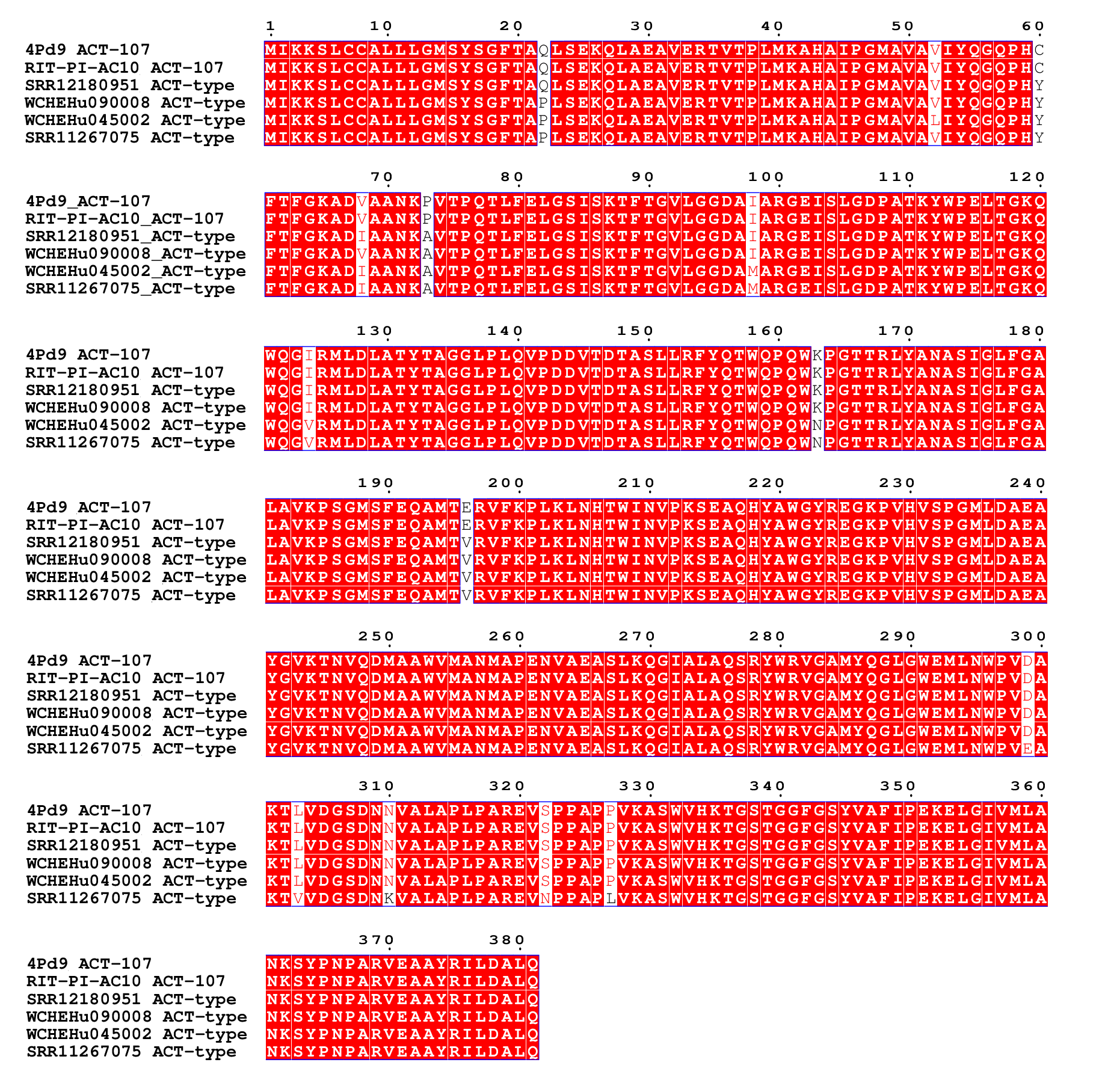
Figure S2**

**Fig S2.** Alignment of amino acid sequences of ACT-107 (UNN26045, this study) and ACT-type from the *E. huaxiensis* available in the NCBI: RIT-PI-AC10 (GenBank accession number: GCA_025642735.1), SRR12180951 (GenBank accession number: GCA_946481955), WCHEHu090008 (GenBank accession number: GCA_003594935.2), WCHEHu045002 (GenBank accession number: GCA_003944645.1), SRR11267075 (GenBank accession number: GCA_945277355.1). Amino acid mutations are surrounded in white boxes.

**
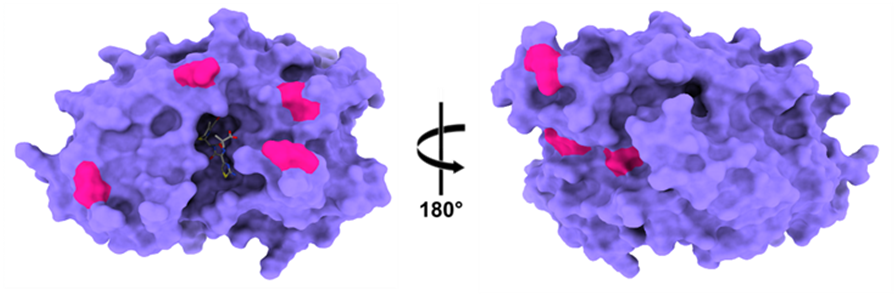
Figure S3**

**Fig S3.** Surface representation of the AlphaFold2 structure model of the ACT-107 AmpC from *E. huaxiensis* strain 4Pd9 complexed to acylated ceftazidime. Position of mutations found in the mature form of the ACT-107 AmpC variant, when compared with all ACT-type β-lactamases publicly available in the beta-lactamase database (BLDB, <http://bldb.eu/>) are highlighted in hot pink.
